# A Neurophysiological Investigation of Non-native Phoneme Perception by Dutch and German Listeners

**DOI:** 10.3389/fpsyg.2016.00056

**Published:** 2016-01-29

**Authors:** Heidrun Bien, Adriana Hanulíková, Andrea Weber, Pienie Zwitserlood

**Affiliations:** ^1^Centre for Psychiatry, Wolfson Institute of Preventive Medicine, Queen Mary University of LondonLondon, UK; ^2^Institute for Psychology, University of MünsterMünster, Germany; ^3^Albert-Ludwigs-Universität FreiburgFreiburg, Germany; ^4^Eberhard-Karls-Universität TübingenTübingen, Germany; ^5^Otto Creutzfeldt Center for Cognitive and Behavioral NeuroscienceMünster, Germany

**Keywords:** L2 substitutions, interdental fricative, Dutch, German, non-native phoneme perception, MMN, ERP

## Abstract

The Mismatch Negativity (MMN) response has often been used to measure memory traces for phonological representations and to show effects of long-term native language (L1) experience on neural organization. We know little about whether phonological representations of non-native (L2) phonemes are modulated by experience with distinct non-native accents. We used MMN to examine effects of experience with L2-accented speech on auditory brain responses. Specifically, we tested whether it is long-term experience with language-specific L2 pronunciations or instead acoustic similarity between L2 speech sounds that modulates non-native phoneme perception. We registered MMN responses of Dutch and German proficient L2 speakers of English to the English interdental fricative /𝜃/ and compared it to its non-native pronunciations /s/ (typical pronunciation of /𝜃/ for German speakers) and /t/ (typical pronunciation of /𝜃/ for Dutch speakers). Dutch and German listeners heard the English pseudoword *thond* and its pronunciation deviants *sond* and *tond*. We computed the identity Mismatch Negativity (iMMN) by analyzing the difference in ERPs when the deviants were the frequent vs. the infrequent stimulus for the respective group of L2 listeners. For both groups, *tond* and *sond* elicited mismatch effects of comparable size. Overall, the results suggest that experience with deviant pronunciations of L2 speech sounds in foreign-accented speech does not alter auditory memory traces. Instead, non-native phoneme perception seems to be modulated by acoustic similarity between speech sounds rather than by experience with typical L2 pronunciation patterns.

## Introduction

Listeners need to correctly discriminate and identify speech sounds in order to succeed in word recognition. There is ample evidence that experience with a given language influences how listeners perceive, discriminate, and categorize speech sounds (Strange, 1995; Cutler, 2012). This can, for example, be seen when looking at discrimination abilities for phoneme contrasts in the listener’s native language (L1) compared to discrimination abilities of unknown contrasts in a second language (L2; e.g., Werker and Tees, 1984). While discrimination in one’s native language is usually easy, discrimination success in a L2 is modulated by how well the non-native sounds fit existing native categories. Indeed, cross-linguistic studies show that different language backgrounds effect L2 speech perception (e.g., Flege, 1995, 2007; Strange, 1995; Best and Tyler, 2007). Models of phonetic perception in L2, such as Flege’s Speech Learning Model (SLM; Flege, 1995) and Best’s Perceptual Assimilation Model (PAM; Best and Tyler, 2007), therefore predict discriminability of phoneme categories by L2 listeners by reference to the relationship of the phoneme repertoires of their first and second language. While PAM deals with inexperienced listeners, Flege’s SLM focuses on experienced L2 learners and predicts increasing difficulties in establishing a new category with a decreasing acoustic-phonetic distance between an L1 and an L2 sound. While neither of these accounts deals with experiential effects from listening to L2-accented speech, they both assign an important role to the phonetic similarity between native and non-native sounds.

Experience also shapes the time course of lexical processing in one’s native language. Listeners recognize words that occur frequently in their L1 more easily than infrequent words (Marslen-Wilson, 1987), they also recognize native pronunciation variants, as in English *corp’rate* for *corporate*, faster when these variant forms are frequent than when they are infrequent (e.g., Ranbom and Connine, 2007; Connine et al., 2008). Such processing advantages for frequent variants are often seen as an indicator for what form might be stored and represented in the mental lexicon (e.g., Ranbom and Connine, 2007). Evidence for experiential effects also comes from cross-linguistic studies examining native and non-native listeners’ processing of frequent L2 pronunciation variants (Hanulíková and Weber, 2012). In their eye-tracking study, English listeners as well as Dutch and German learners of English differed in the recognition speed of English words in which the initial phoneme /𝜃/ was substituted by /s/, /f/, or /t/ (e.g., *theft* pronounced as /t𝜀ft/, *theme* as /fi:m/, and *thrill* as /sril/). In a production experiment, Hanulíková and Weber (2010) showed that while /t/, /f/, and /s/ are the three most common /𝜃/-substitutions, the relative frequency with which they occur differs across the Dutch and German speakers’ non-native productions. The dominant /𝜃/-substitute for German speakers is /s/, while for Dutch speakers it is /t/. Eye-tracking data from Hanulíková and Weber (2012) revealed that recognition ease of non-native variants reflects these distinct production patterns. For example, listeners heard *theft* pronounced as the variant /t𝜀ft/ and saw four printed words on a computer screen: the intended English word (e.g., *theft*), a phonological rhyme competitor (e.g., *left*), and two unrelated distracters (e.g., *kiss* and *mask*). Looking preferences for target words (e.g., the printed word *theft*) matched the language-specific preferences for producing these variants. Dutch listeners fixated the target words most often when hearing variants with the /t/-substitutions, and German listeners did so when hearing the /s/-substitutions. The authors concluded that linguistic experience with L2 pronunciations facilitates recognition of these variant forms in L2 listening. As robust as these effects are, it remains unclear whether they originate from a phonemic or lexical level.

While experiential factors in L1 perception have been well studied, very little is known about the consequences of L2 experience for neural representations of L2 phonemes. Does experience with typical pronunciations of L2 speech sounds lead to cross-linguistically distinct memory traces for non-native phonemes? The goal of the present study is thus to investigate the effects of long-term L2 experience on the nature of phoneme categories of a second language. To this end, we measured auditory brain responses by using the Mismatch Negativity (MMN). Specifically, does language-specific experience, due to the frequency of pronunciation variants of the English voiceless interdental fricative /𝜃/, result in distinct MMN responses to /𝜃/-substitutions as a function of specific differences between Dutch and German accented speech?

### Production and Perception of English Interdental Fricatives

The English fricative /𝜃/ presents great difficulties in production for many learners of English, and even highly proficient L2 learners regularly substitute English /𝜃/ with other sounds, most often /s/, /t/, and /f/ (for an overview, see Brannen, 2002). The preferences for substitutes depend on the L1 background of L2 speakers (e.g., Brannen, 2002; Hanulíková and Weber, 2010). Hanulíková and Weber (2010) have shown that German learners of English commonly substitute /𝜃/ with /s/ (29%) and to a much lesser extent with /t/ (7%) or /f/ (5%), while Dutch learners prefer to use /t/ (23%) and to a much lesser extent /s/ (5%) or /f/ (3%); (Note that all three substitutes are phonemes of both Dutch and German). As a consequence, it is reasonable to assume that German learners experience /s/-substitutes (as in /s𝜀ft/ for *theft*) the most, while Dutch speakers are most often presented with /t/-substitutes (as in /t𝜀ft/ for *theft*). In the present study, we therefore focus on the perception of these two most frequent substitutes.

/𝜃/ and /s/ are acoustically slightly more similar than /𝜃/ and /t/. From an articulatory viewpoint, /𝜃/ and /s/ are fricatives, realized with a constriction in the oral tract that causes turbulent airflow. /t/ on the other hand is an oral stop consonant, for which the vocal tract is first blocked, stopping all airflow, before it is released with a burst. /𝜃/ is characterized by a relatively flat spectrum with no clearly dominating peaks, while alveolar /s/ displays an intense primary spectral peak at higher frequencies (e.g., Jongman et al., 2000). The spectrum of /t/ has a diffuse spread of energy, with peak amplitudes being larger in the high frequencies (e.g., Stevens and Blumstein, 1978). The two groups of Dutch and German L2 learners of English are particularly interesting, because they not only differ in their predominant [𝜃]-substitutions, but also in the acoustic properties of both /s/ and /t/ in their respective L1 and from their L2 English. Dutch /s/ is less articulatorily tense and has graver friction than German or English (Mees and Collins, 1982; Rietveld and van Heuven, 2001; Hanulíková and Weber, 2010), and /t/ in initial position is aspirated in German (and in English) but unaspirated in Dutch (Lisker and Abramson, 1964; Keating, 1984).

These acoustic similarities and differences between /𝜃/-/s/ and /𝜃/-/t/ do not necessarily affect the ability to perceptually discriminate these pairs. Oﬄine discrimination and identification tasks show that non-native listeners can distinguish between /𝜃/ and /s/ and /𝜃/ and /t/ quite well (e.g., Hancin-Bhatt, 1994; Cutler et al., 2004; Hanulíková and Weber, 2012). For example, Cutler et al. (2004) have found that Dutch L2 listeners confuse English /𝜃/ (in 0-db SNR) with /t/ 6.3% and with /s/ 0.4%. Hancin-Bhatt (1994) showed that German listeners in good listening conditions misidentify /𝜃/ as /t/ 0% and as /s/ 5%. In line with this pattern, Hanulíková and Weber (2012) showed in an AXB task that performance for both /𝜃/-/s/ and /𝜃/-/t/ contrasts was high and comparable across Dutch and German listeners (on average 89% correct for the /𝜃/-/s/ contrast and 90% correct for the /𝜃/-/t/ contrast). Although Dutch and German listeners can perceptually distinguish between /𝜃/-/s/ and /𝜃/-/t/ quite well, their productions show clear preferences toward one of the variants. While these production preferences affect lexical processing, it is less clear whether non-native phoneme perception is affected as well. This raises the question of the level at which such experiential effects arise during processing. Does the frequency of production variants in L2 speech already affect pre-attentive processing of speech sounds at a pre-lexical level? In other words, is the memory representation of /𝜃/ closer to /s/ for German listeners, and to /t/ for Dutch listeners?

### MMN Studies on Effects of Experience in Speech Perception

An excellent tool to investigate experience-based auditory memory traces is Mismatch Negativity, an early event-related brain potential (ERP) generated in the auditory cortex. It is a negative ERP component that occurs between 150 and 350 ms after the detection of a deviant feature in the stimulus. In a typical MMN design, the so-called standard stimulus (sound, syllable, or word) is presented 80–90% of the time while the so-called deviant stimulus (sound, syllable, or word) is presented 10–20% of the time. It is assumed that the MMN is evoked through a mismatch of the properties of a deviant stimulus and the neural traces in sensory memory consigned by the repeated presentation of a standard stimulus, irrespective of the direction of the subject’s attention or task. As such, an MMN design allows the examination of amplitude differences upon the detection of a change between standard and deviant pronunciations.

Since its discovery in the 1970s (Näätänen et al., 1978), MMN has been linked to various aspects of deviant acoustic properties (for an overview, see Shtyrov and Pulvermüller, 2007) such as pitch (e.g., Näätänen and Gaillard, 1983; Jacobsen et al., 2003), stimulus duration (Paavilainen et al., 1991), and loudness (e.g., Keidel and Spreng, 1965). It has been shown that better discrimination of a native or a non-native phonetic contrast is reflected by higher MMN amplitudes (e.g., Winkler et al., 1999; Shafer et al., 2004). Näätänen et al. (1997) were among the first to observe such language-specific phoneme representations using MMN. In their study, Finnish and Estonian participants were presented with the vowel /e/ (used as standard in the MMN design), that is present in both languages, as well as with vowels /ö/, /o/, /õ/ (used as deviants in the MMN design), of which the first two are present in both languages but the last one only exists in Estonian. Näätänen et al. (1997) found that the amplitude of the MMN was influenced by the deviant’s phonemic status in the respective language. There was larger MMN for vowels that were present in the participant’s native language (Finnish) compared to vowels that were not present. The effect did not seem to be affected by acoustic features, since the deviant vowels were equally complex. Larger MMN occurred only when the deviant stimulus was part of the respective phoneme inventory. This result led to the suggestion that memory traces of speech sounds are language-dependent, and reflect native phoneme categories (cf. Bien and Zwitserlood, 2013). A replication of the result came from data from 12-months-old but not from 6-months-old infants, suggesting an early development of language-specific memory traces (Cheour et al., 1998).

In the same line of research, Dehaene-Lambertz (1997) found that native French-speaking subjects display MMN when confronted with an acoustic change signaling a phonemic boundary in French but not in Hindi. Effects of experience with a L1 are also visible when experience is operationalized as the relative frequency of occurrence for a given phonological process in a given context. To examine sensitivity to frequency of phonological variants, Tavabi et al. (2009) created German bisyllables and manipulated the phonemic context in which assimilation of /n/ to /m/ occurs (e.g., *onbo* to *ombo*) as well as the frequency of assimilation (/n/ to /m/ is more frequent than /m/ to /n/). They found that both the frequency of the particular assimilation and the context in which it occurs modulate the MMN.

MMN can be used to index the perception and discrimination abilities of foreign-language phonemes as well; Winkler et al. (1999) demonstrated that Hungarian participants with no prior exposure to Finnish showed no MMN and very poor discrimination performance with the Finnish vowel contrast (/e/ – /æ/). Hungarians who were fluent in Finnish showed a MMN that was comparable to the one found in native Finnish-speaking participants. Training effects for the perception of non-native contrasts – Germans learning moraic consonant duration in Japanese – are reflected in the emergence of an MMN (Menning et al., 2002). Interestingly, presenting a continuum of synthesized Hindi stops to English and Hindi speakers, Shafer et al. (2004) found some evidence that pre-attentive discrimination is modulated by experience with the speech sounds of a language. The observed MMN did not, however, directly correspond to the behavioral discrimination results, and some pairs of sounds that could be behaviorally discriminated did not elicit MMN. Long-term experiential factors with L2 phoneme duration were tested by Nenonen et al. (2003). They found that – despite extensive experience with the L2 and advanced L2 skills – non-native listeners did not reach native-like discrimination abilities for speech stimuli (but they were comparable with natives when tested with non-speech stimuli).

Taken together, these studies suggest that the MMN can be used as an index of long-term experience with native and non-native speech sounds, using single speech sounds, syllables, and non-words. In this study, we examine electrophysiological activity of the brain to understand the perception of English dental fricative sounds in two groups of proficient L2 listeners, which has been rarely done. Some previous research (mainly using magnetoencephalography) on fricative perception examined L1 English phonemic contrasts such as /s/ and ∫ (Miller and Zhang, 2014; Lago et al., 2015) as well as responses to Polish fricatives by native and inexperienced non-native listeners (Lipski and Mathiak, 2007). It remains unclear whether experience with typical mispronunciations of L2 speech sounds lead to cross-linguistically distinct memory traces for non-native phonemes. In our study, we examined this question by using English monosyllables with no lexical status to avoid possible top-down effects (Pulvermüller and Shtyrov, 2006), and to focus on L2 memory traces for phonemes.

### Present Study

In the present study, we use MMN to look at the role of experience with common mispronunciations in a second language. Specifically, we examine whether cross-linguistically distinct experience with mispronunciations of L2 speech sounds shapes the neural organization of L2 phonemes, as reflected in the size of mismatch effects. Studying Dutch and German participants, we focus on the perception of the voiceless interdental fricative /𝜃/ and its substitutions /t/ and /s/, most commonly produced by these two groups of learners of English.

To examine the influence of experience with non-native accents on auditory memory traces, we compared the automatic electrophysiological responses in Dutch and German listeners to the English pseudoword *thond* and its pronunciation variants *sond* and *tond* in an oddball paradigm. Oddball paradigms are not free from lexical effects, even with attention diverted from the acoustic stimuli (cf. Pulvermüller and Shtyrov, 2006). Therefore, we used English monosyllabic pseudowords. We concentrated on the variant forms /s/ and /t/ as they represent the preferred substitute for the two learner groups respectively. If long-term experience with typical non-native variants already affects this early automatic processing level, we should find a similar accent-specific pattern of results as reported for lexical processing in Hanulíková and Weber (2012). That is, smaller mismatch effects should be found for *tond* than for *sond* for Dutch listeners, and the reverse should be found for German listeners. Alternatively, exposure-frequency effects might only arise at higher levels of lexical processing and might not affect non-native phoneme representations. In that case, the two variant forms *tond* and *sond* might either elicit comparable brain responses, or they might reflect effects of stimulus similarity, in which case *tond* should elicit a larger mismatch effect than *sond* for both Dutch and German participants.

## Materials and Methods

### Participants

Eighteen native speakers of Dutch (mean age: 23, *SD*: 3.3, nine male) and 17 native speakers of German (mean age: 23, *SD*: 1.6, three male) participated in the present study, after having given written, informed consent. Dutch participants were tested in the Netherlands, at the Max Planck Institute for Psycholinguistics. German participants were tested in Germany, at the University of Münster. All participants reported having normal hearing and no history of neurological problems, head injuries, or continuous medication. Participation was compensated with €12 or course credit.

Subsequent to the experiment, participants took part in an ABX discrimination test of the speech materials, and provided information on their use of and proficiency in English. All German participants had learned English in school as their second language with a mean duration of 8.4 years (*SD*: 0.8). Dutch participants had on average 7.6 years (*SD*: 0.7) of English education in school. In the Netherlands, all students in upper educational levels have to attend German language courses for at least 3 years, and German is usually their third or fourth non-native language (after English). Thus, all Dutch participants had some knowledge of German. Dutch, on the other hand, is not mandatory in German high schools, and German participants had little or no exposure to Dutch.

This study was carried out in accordance with the recommendations for ethical guidelines of the Institute for Psychology, Westfälische Wilhelms-Universität, Münster, Germany and Max Planck Institute for Psycholinguistics, Nijmegen, The Netherlands. All participants gave written informed consent in accordance with the Declaration of Helsinki.

### Stimuli and Design

**FIGURE 1:**
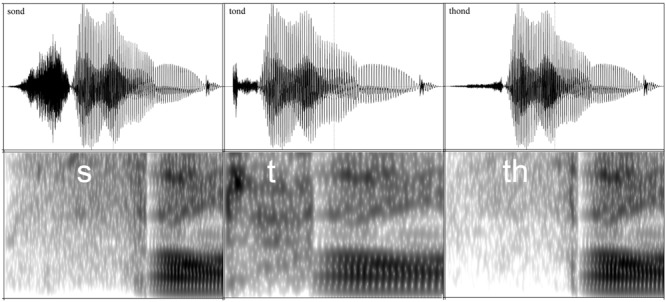
**Waveforms of stimuli and spectrograms of the initial consonants /t/, /s/, /𝜃/ with a 90 ms portion of the following vowel /o/.** Frequencies are shown from 0 to 5 kHz on the horizontal axis.

We compared processing of the English interdental fricative /𝜃/ in the pseudoword *thond* with the non-native pronunciation variants *tond* and *sond*. The variant *sond* represents a typical pronunciation of *thond* for German speakers of English, who frequently substitute /𝜃/ with /s/, while the variant *tond* is typical for Dutch speakers of English, who frequently substitute /𝜃/ with /t/ (cf. Hanulíková, and Weber, 2010). The stimuli used in the experiment were therefore the English monosyllabic pseudowords *thond, sond*, and *tond*. To ensure a native-like pronunciation of /𝜃/ in *thond*, all pseudowords were produced by a native speaker of English. None of the stimuli is, or closely resembles, an existing word in Dutch or in German. In addition, pronounced as English pseudowords, *thond, sond*, and *tond* cannot be interpreted as Dutch or German pseudowords, due to a violation of the phonotactic constraint of syllable-final devoicing (e.g., in Dutch and in German, the pseudoword *sond* would be pronounced /sont/). The length of the initial consonants was 149 ms for *thond*, 60 ms for *tond* and 176 ms for *sond*. The length of the stimuli was 593 ms for *thond*, 499 ms for *tond*, and 609 ms for *sond*. The stimuli were cross- and identity-spliced to avoid elicitating MMN due to features other than the initial phoneme in the recorded materials (see **Figure 1** for stimuli waveforms and spectrograms after the splicing procedure). Some variation in the stimuli was re-created by changing the pitch to abstract away from specific acoustic properties of individual tokens (e.g., Bien et al., 2009). With three stimuli and five levels of pitch (+12, +6, +0, -6, and -12 Hz), the total number of tokens was 15. All stimuli served as both standards and deviants in different blocks.

The experiment consisted of four blocks, each with a different STANDARD_deviant combination ([THOND_tond]; [THOND_sond]; [TOND_thond]; [SOND_thond]). The order of blocks was balanced across participants. Within each block, 500 stimuli were presented in random order, with a deviant likelihood of 20% and an inter-stimulus interval of 1000 ms. Each block lasted for approximately 11 min, and there was a short break after each block. The experimenter started the next block once the participants had retaken a stable and comfortable position.

### Procedure and EEG Recording

Participants were comfortably seated in front of a computer screen in a sound-attenuated room. The stimuli were presented via loud speakers at approximately 60 dB SPL. During the electrophysiological recordings, participants watched a silent movie and were told that they could ignore the auditory stimuli.

The electroencephalography (EEG) of the German participants (GER) were recorded in sampling rates of 256 Hz, using 64-channel WaveGuard caps (ANT, Enschede, Netherlands) connected to an ANT amplifier (ANT, Enschede, NL). AFz was used as the ground electrode, and electrode impedances were kept below 5 KΩ. Horizontal eye movements were recorded using two bipolar electrodes with left and right canthal montage. Lateral eye movements and blinks were recorded using two bipolar electrodes placed above and below the right eye. An average mastoid reference was used.

Electroencephalography for the Dutch participants (NL) was recorded from 34 Ag–AgCl electrodes (Brain Products, MedCat, Netherlands) at standard 10–20 locations. Impedance was kept below 5 kΩ. All recordings were referenced to the left mastoid during recording (eye movement and blink artifacts were recorded from F9 to F10 and from Fp1 to an additional EOG electrode below the left eye), amplified with BrainAmp DC amplifiers (0.016–100 Hz band pass, digitized at 500 Hz), and re-referenced off-line to the mastoid average (e.g., Poellmann, 2013; Poellmann et al., under revision).

Data were analyzed with Advanced Source Analyses (ASA) software (ANT Software BV, Enschede, NL) and with SPSS statistics. We filtered the data oﬄine, applying a 35 Hz low-pass filter. EEGs outside the range of -75 to +75 μV were labeled as artifacts and excluded from further analyses. This ensured the elimination of segments containing eye movement, blinking, or muscular activity. Overall, 81% of epochs were free from artifacts and used for analyses (71% for German and 91% for Dutch participants). Intact epochs were evenly distributed across conditions within each group. The remaining data were averaged in epochs of 800 ms, including a 250 ms pre-stimulus baseline interval used for epoch correction. All analyses were based on the mean amplitudes at Cz.

**FIGURE 2:**
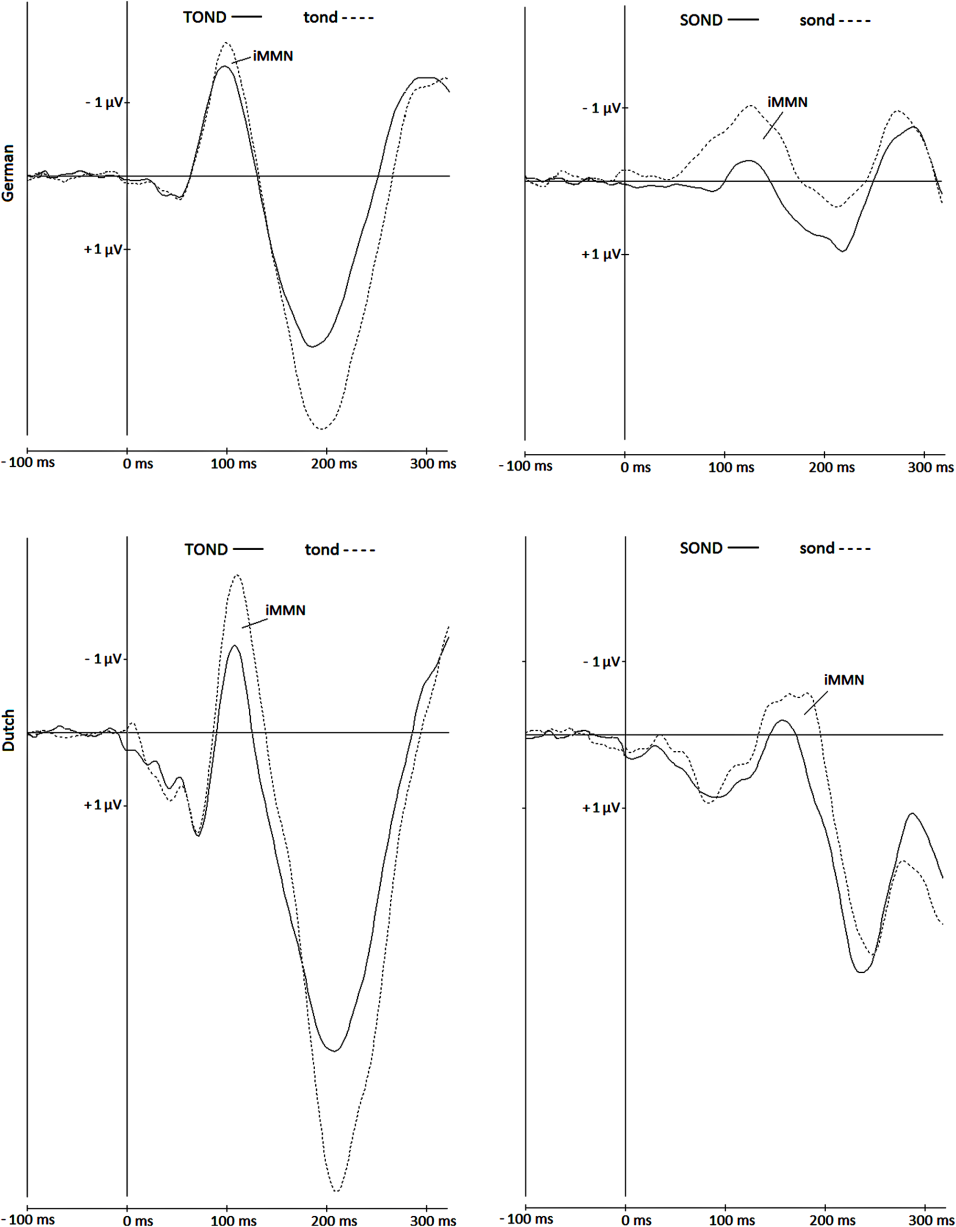
**iMMN: the grand averages at Cz, elicited by *tond***(left)** and *sond***(right)**, presented as standard (solid line) and as deviant (dotted line) next to *thond*, in German (upper graphs) and Dutch (lower graphs) participants**.

We analyzed the *identity* mismatch (*i*MMN) elicited by the *thond*-pronunciation deviants *tond* and *sond* in the Dutch and German participants. In order to compute the *i*MMN for *tond* and *sond*, we subtracted the respective ERPs when used as a standard next to *thond* from the ERPs when used as a deviant next to *thond*. That is, for the iMMN of *tond*, the standard-ERP elicited by *tond* in the block [TOND_thond] was subtracted from its deviant-ERP elicited in the block [THOND_tond]. For *sond*, the standard-ERP in block [SOND_thond] was subtracted from its deviant-ERP elicited in block [THOND_sond]. This *i*MMN procedure is specifically relevant when stimuli differ with respect to duration and various spectral factors, which certainly holds for fricatives and plosives. Calculating iMMN cancels out the specifics of the individual acoustic stimulus tokens (**Figure 2**).

Based on qualitative visual inspection, the time windows for the analyses of the mismatch effects were determined by the range of the deviant-N1. Note that mismatch negativity often overlaps with the N1 (cf. Schröger, 1998). The data-driven selection of the time windows was done separately for the Dutch and German groups of listeners. Measured at Cz (**Figure 2**), where both the N1s and the mismatch effects were most prominent, the time windows were 88–140 ms (Dutch) and 68–133 ms (German) for *tond*, and 134–196 ms (Dutch) and 78–168 msec (German) for *sond*. The use of different time windows is justified for the factor *phoneme* because of the large variance in the onset of perceivable information between the critical stimuli. Likewise, because listeners’ perception is optimized for their native language, Dutch and German listeners differ with respect to the uptake of information that distinguishes between phonemes (the voice-onset times vs. prevoicing distinction for voiced plosives is a prime example). Thus, to select most objectively for the planned comparison of the identity mismatch elicited by a given stimulus, we also opted for data-driven (and thus potentially different) time windows for the factor *listener group*. Note that it is not uncommon to use data-driven solutions to the problem of latency variability (see Luck, 2005, p. 135). All analyses were based on the mean amplitudes at Cz within the specified time windows (i.e., over the whole range of the deviant-N1). We followed the suggestions by Luck (2005) to use an area amplitude measure rather than a peak amplitude measure to mitigate the reduction in amplitude caused by latency variability amplitude (see also Schröger, 1998). For the statistical analysis, we used a 2 (Deviance: sond, tond) by 2 (Group: Dutch, German) repeated-measures analyses of variance (ANOVA) and report Greenhouse–Geisser corrections and corrected F-values where appropriate. Additionally, we report one-way ANOVAs for Deviance for each group separately.

### Behavioral ABX-Experiment

After the EEG experiment, all participants completed a speech-sound discrimination ABX-task to test participants’ discriminatory ability for the three stimuli. Stimuli /𝜃ond/, /tond/, and /sond/ were presented over speakers in 12 trials in a random order at the A and B positions, followed by a third stimulus at the X position that matched either A or B. Participants had to press the left shift button when the last presented (X) stimulus matched the first (A) stimulus, and to press the right shift button when it matched the second (B) stimulus. Stimuli were presented at ISIs of 800 ms.

The results showed that German participants distinguished /𝜃ond/ equally well from /tond/ (2,9% errors) and /sond/ (4,4% errors). For Dutch participants, it was harder to distinguish /𝜃ond/ from /sond/ (22,2% errors) than from /tond/ (8,3% errors). A closer look at the Dutch participants shows that the higher error rate is mainly due to three participants. The error rate drops to 9,3% when these participants are excluded. Note that this discrimination pattern would go against the predicted experience-based perception effect in the EEG study, according to which Dutch speakers would perceive /t/ as a closer match to /𝜃/ than to /s/ (as reflected in their production behavior). Moreover, previous studies reported non-significant MMN responses to L2 contrasts in L1 and L2 participants despite the presence of differences in behavioral discriminations of L2 contrasts (e.g., for the Japanese listeners’ difficulties with the English /r/ and /l/; Zevin et al., 2010).

## Results

Visual inspection of the data in **Figure 2** indicated that, when presented next to *thond*, both pronunciation variants *sond* and *tond* elicited mismatch effects in both groups of participants. However, against our hypothesis, the numerical differences of mismatch effects for /s/ and /t/ between the language groups show larger effects for /tond/ in Dutch listeners, and larger effects for /sond/ in German listeners.

In a first step, we tested the significance of each identity mismatch component against zero in each group of participants. Subtracting the standard-ERP of *sond*, elicited in block [SOND_thond], from its deviant-ERP, elicited in block [THOND_sond], the identity mismatch effect (mean amplitude in the specified time window) was -0.65 μV [*t*(1,17) = 4.51, *p* = 0.049] for Dutch participants and -0.81 μV [*t*(1,16) = 7.08, *p* = 0.017] for German participants. The difference in the *sond*-*i*MMNs between the two groups was non-significant (independent sample *t*-test [*t*(33) = 0.379, *p* = 0.707]). *Tond* elicited iMMNs of -0.93 μV [*t*(1,17) = 8.31, *p* = 0.010] in Dutch participants and -0.20 μV [*t*(1,16) = 0.44, *p* = 0.519] in German participants. The difference in the *tond*-*i*MMNs did not reach significance (independent-sample *t*-test [*t*(33) = -1.667, *p* = 0.105]).

In a second step, ANOVAs on the identity mismatch effects were carried out separately for the two groups of listeners; the factor Deviance (*sond, tond*) was not significant in either the Dutch [*F*(1,17) = 0.35, *p* = 0.563] or German group [*F*(1,16) = 1.88, *p* = 0.189]. An overall ANOVA with Group (Dutch, German) as the between-subjects factor and Deviance as the within-subjects factor revealed no main effect of Deviance [*F*(1,33) = 0.027, *p* = 0.609], no main effect of group [*F*(1,33) = 0.961, *p* = 0.334], and no significant interaction between Group and Deviance [*F*(1,33) = 1.88, *p* = 0.180; see **Table 1**].

**Table 1:** Analyses of variance on the identity mismatch effects (mean amplitudes in μV) elicited by the *thond*-pronunciation variants *tond* and *sond*, computed with GROUP (Dutch, German) as a between-subject factor, and for each group separately.

Dutch listeners DEVIANCE (*sond, tond*) *tond*-0.93 μV *sond*-0.65 μV	*F*(1,17) = 0.35 *t*(17) = 8.31 *t*(17) = 4.51	*p* = 0.563 *p* = 0.010 *p* = 0.049
German listeners DEVIANCE (*sond, tond*) *tond*-0.20 μV *sond*-0.81 μV	*F*(1,16) = 1.88 *t*(16) = 0.44 *t*(16) = 7.08	*p* = 0.189 *p* = 0.519 *p* = 0.017
Overall GROUP (Dutch, German) DEVIANCE (*sond, tond*) DEVIANCE ^∗^ GROUP	*F*(1,33) = 0.96 *F*(1,33) = 0.27 *F*(1,33) = 1.88	*p* = 0.334 *p* = 0.609 *p* = 0.180

To summarize, mismatch effects were seen for both deviant stimuli in both language groups (except for the iMMN for *tond* in the German group, where it was expected to be pronounced). Interestingly, the pattern of identity mismatch elicited by the *thond-*pronunciation variants *sond* and *tond* was comparable in both groups of listeners (see **Table 1**). This does not confirm an L2-accent-specific pattern of results, according to which smaller mismatch effects were expected for *tond* than for *sond* for Dutch listeners, and the reverse was expected for German listeners. What we observed instead is that the two variant forms *tond* and *sond* elicited comparable brain responses across the two listener groups and thus might reflect effects of stimulus similarity.

## Discussion

The present study examined whether pre-attentive processing of pronunciation variants in non-native speech is influenced by cross-linguistically distinct experiences with such variants. If experience exerts a predominant influence on speech processing and speech-sound representation, smaller mismatch effects were expected for *tond* compared to *sond* in Dutch listeners, for whom /t/ is the common substitute for /𝜃/ (Hanulíková and Weber, 2010). The reverse was expected for Germans, who frequently substitute /𝜃/ with /s/ (Hanulíková and Weber, 2010). While there is converging evidence that experience with pronunciation variants in an L2 influences speech processing at a lexical level (e.g., Hanulíková and Weber, 2012), the present study found no evidence for an impact of experience with L2 pronunciations on L2 phoneme representations. We did not find (at least in the ANOVA analysis) the predicted differential processing between the two groups. Note that the numerical differences of mismatch effects for /s/ and /t/ between the language groups were even against the hypothesized direction, with larger effects for /tond/ in Dutch listeners, and for /sond/ in German listeners. A possible explanation is provided further below, however, given the lack of interaction, these differences should not be interpreted.

In the ERPs of Dutch and German proficient speakers of English, we compared the identity mismatch effects elicited by the pronunciation variants *sond* and tond in the context of the English pseudoword *thond*. Presented next to the pseudoword *thond*, the pronunciation variants *sond* and *tond* elicited mismatch effects in both the Dutch and German groups of listeners. This was evident when using the identity mismatch approach (presenting two blocks with switching roles of standard and deviant for each variation of interest, then subsequently comparing the ERPs elicited by the identical stimulus when presented as standard and when presented as deviant).

Due to its clearer acoustic onset, *tond* elicited a more distinct and more negative N1 than *sond*, both when presented as a standard and when presented as a deviant. This acoustic difference, however, was not confirmed in a main effect of Deviance in the iMMN. Most importantly, the mismatch effects elicited by *sond* and *tond* were statistically comparable, and there were no interactions with listener group. This result seems to reflect effects of stimulus similarity comparable across both listener groups. Note that we used English monosyllabic pseudowords (*thond, tond, sond*) and avoided lexical items because pronunciation substitutions may vary depending on the phonemic context and position in a given word. An interesting question for future research is thus whether the same result would be obtained for real words.

It should be noted, however, that while the mean latencies of the time frames were similar between the two listener groups in the *tond*-condition, they were different in the *sond*-condition. One possible reason for this difference could stem from a different uptake of acoustic cues for the English fricative between the two listener groups. Previous studies have shown that Dutch /s/ is articulatorily less tense and has graver friction than German /s/ (Mees and Collins, 1982) and therefore also differs from English to a larger extent than German (e.g., Hanulíková and Weber, 2010, 2012). Slight latency differences present in the *tond*-condition could also be explained by the distinct L1 acoustic characteristics. The Dutch /t/ is less aspirated than the English /t/ and mainly uses prevoicing as a cue to voicing, while German listeners use the VOT to categorize voicing of plosives (Lisker and Abramson, 1964; Keating, 1984). This could lead to distinct /t/-categorization patterns for German compared to Dutch listeners. These differences could have perceptual consequences and this could explain why the mapping of the English /s/ and /t/ sounds onto the distinct Dutch /s/ and /t/ resulted in different latencies than mapping of the English sound onto the more similar German /s/ and /t/. Distinct uptake processes of acoustic information are very likely despite behavioral tasks suggesting that both Dutch and German listeners show comparably high discriminatory performance for both /𝜃/-/s/ and /𝜃/-/t/ contrasts (e.g., Hanulíková and Weber, 2012). Although the control ABX test run after the present EEG study showed a higher error rate for /𝜃/-/s/ compared to /𝜃/-/t/ in the group of Dutch participants, this was mainly due to three participants. It would be interesting for future research to examine the issue of how cues are weighted differently in the foreign and native languages, particularly in the two highly related languages such as German and Dutch.

Finally, the time windows for analyzing the auditory components were selected based on the grand average of the deviant-N1s. Though we selected the time windows separately for *tond* and *sond* and separately for the group of Dutch and German participants, selection was not based on the individual responses of each participant. The time spans of certain ERP components can vary greatly across individuals (e.g., Michaelewski et al., 1986). As a consequence, group average analyses can eliminate individual mismatch effects, underestimating their actual size. The observation of significant effects based on group averages can be considered a strong indicator that these effects are real. Moreover, we restricted our analysis to the first N1 elicited by the speech sound, time-locked to its onset. Another approach would be to also analyze the second N1, elicited by the acoustic change complex defined as – and time-locked to – the transition of the consonant to the following vowel (see Lipski and Mathiak, 2007; Miller and Zhang, 2014). The ERP data in both subject groups (see **Figure 2**) indicate the consistent elicitation of the negative deflection responses to fricatives. Future research might want to focus on the time-point-by-time-point ERP responses to the speech stimuli to accommodate the complexity of ERP waveforms that could arise from tracking the acoustic properties of speech stimuli in the time domain.

A number of MMN studies found language-specific effects on phoneme perception (e.g., Dehaene-Lambertz, 1997; Näätänen et al., 1997; Winkler et al., 1999; Jacobsen and Schröger, 2003; Nenonen et al., 2003). However, most of these studies examined native listeners’ perception or L2 perception of familiar and unfamiliar contrasts with within- or across-category manipulation for a given language. The present study used phonemes that are frequently produced by non-native speakers of English as substitutions for interdental fricatives. The present study is thus the first to look at whether cross-linguistically distinct experience with frequent non-native pronunciations modulates phoneme representations. The results suggest that experience-based memory representations for frequent or preferred phoneme substitutions are not (or not well) established. Given previous research on differences between native and advanced learners’ perception of acoustic features (e.g., Nenonen et al., 2003), the present result may not be surprising. The formation of stable language-specific memory representations in an L2 seems to require early exposure or, for late learners, frequent exposure and practice. Listeners can learn to distinguish non-native contrasts, even when they are unable to produce these (Menning et al., 2002). It would be good if learning novel L2 speech sounds would result in the formation of a new perceptual category (for /𝜃/, for example), even if correct production lags behind. The use of L2 pronunciation variants may facilitate lexical processing, but does not lead to an annexation of novel sounds into native categories. Indeed, cross-linguistic perception studies and models (e.g., SLM, PAM) suggest that it is more difficult to establish a new L2 phonological category when the acoustic-phonetic properties of an L2 sound are similar to an L1 sound (Flege, 1995, 2007; Best and Tyler, 2007; see Dobel et al., 2009, for evidence from the N400 component). While these models are not directly concerned with L2-accented speech, the present results could be explained on the basis of acoustic/auditory properties of the consonant pairs (/𝜃/ as more similar to /s/ than to /t/).

Taken together, the results suggest that long-term non-native experience with frequent pronunciation variants in a second language does not alter memory traces of phonemes or perception of these L2 speech sounds. Instead, pre-attentive perception of non-native speech sounds may better be explained in terms of acoustic similarity to native categories.

## Author Contributions

All authors listed, have made substantial, direct and intellectual contribution to the work, and approved it for publication.

## Conflict of Interest Statement

The authors declare that the research was conducted in the absence of any commercial or financial relationships that could be construed as a potential conflict of interest.
